# Challenges and Opportunities in Smart Biosensing for Biomanufacturing

**DOI:** 10.1021/acssynbio.5c00203

**Published:** 2025-08-22

**Authors:** Mara Pisani, Pablo Carbonell

**Affiliations:** † Synthetic and Systems Biology Lab for Biomedicine, Instituto Italiano di Tecnologia-IIT, Largo Barsanti e Matteucci, 80125 Naples, Italy; ‡ Open University Affiliated Centre, Milton Keynes MK9 1FW, U.K.; § Institute for Integrative Systems Biology I2SysBio, Universitat de València-CSIC, Escardino Street 9, 46980 Paterna, València, Spain

**Keywords:** biosensor, dynamic regulation, high-throughput screening, computer-in-the-loop

## Abstract

Traditional metabolic engineering has largely focused on the direct construction of synthetic metabolic pathways, often overlooking the critical role of regulation. In contrast, natural metabolic pathways are inherently tightly regulated, enabling robust performance in dynamic environments. Dynamic regulation of synthetic metabolic pathways enhances the reliability of cell factories by improving their performance and ensuring greater robustness, scalability, and stability. Therefore, modern approaches to metabolic engineering should embrace genetic circuits that incorporate dynamic regulatory mechanisms. Biosensors, as key components of these circuits, not only enable precise genetic regulation but also provide real-time monitoring and external interfacing capabilities with diverse signal modalities, including electrical and optical systems. By the incorporation of dynamic control mechanisms, synthetic pathways can be rendered more robust to environmental fluctuations during scale-up and more precisely regulated in therapeutic contexts, such as responsive drug delivery. These capabilities are critical to advancing the reliability and applicability of engineered metabolic systems. Furthermore, the potential for the external control of synthetic metabolic processes, guided by advanced algorithms, underscores the growing importance of machine learning and data-driven approaches. This perspective highlights the necessity of integrating regulation into synthetic pathways and leveraging biosensors to drive the next generation of scalable and adaptive metabolic engineering solutions.

## Graphical abstract

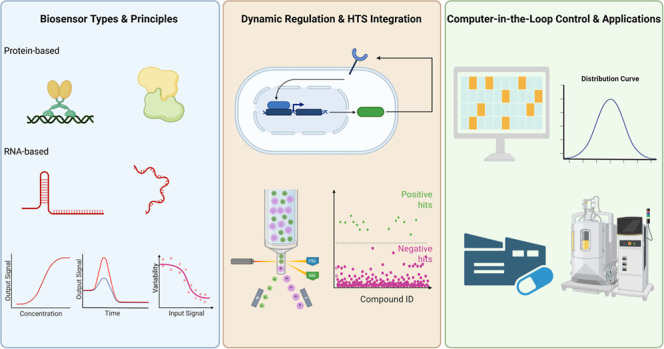

## Introduction

Sensing within cells is a fundamental process that has naturally evolved to provide organisms with an evolutionary advantage, enabling precise responses to dynamic internal and external environments. Different types of biosensors, such as transcription factors, RNA-based systems, protein-based systems, and two-component signaling pathways, allow cells to detect and respond to a broad array of signals, including metabolite levels, ion concentrations, pH, and other biochemical indicators.^1^ Biosensors are fundamental biological components that combine a sensor module, which detects specific intracellular or environmental signals, and an actuator module, which drives a measurable or functional response. These sensing strategies maintain metabolic homeostasis and adaptability under fluctuating conditions, forming the basis for many natural regulatory networks.

Other key performance metrics for biosensors include:

1. Dynamic Range, the span between the minimal and maximal detectable signals
2. Operating Range, the concentration window where the biosensor performs optimally
3. Response time, the speed at which the biosensor reacts to changes
4. Signal-to-noise ratio, the clarity and reliability of the output signal

These fundamental elements are essential for designing effective biosensors for metabolic engineering and synthetic biology.

In metabolic engineering, traditional approaches have primarily focused on enhancing DNA sequences and optimizing microbial chassis to improve the transcription and translation efficiency. However, feedback regulation, a critical component of natural metabolic systems, has often been overlooked. This oversight contrasts sharply with nature’s reliance on intricate regulatory networks to ensure the robustness and stability of biosynthetic pathways under varying conditions.^2,3^

Dynamic control circuits in biosensors are typically characterized by input–output dose–response curves, but their dynamic performance—such as the response speed and signal-to-noise ratio—should also be key metrics of their evaluation. Traditional performance metrics, such as the dynamic range and operating range, remain essential in the biosensor design, but for applications requiring precise and rapid regulation, the ability to quantify response times and manage signal noise is becoming increasingly important. For example, slow response times can hinder controllability, introducing delays in critical processes. To address these limitations, hybrid approaches that combine slower, stable systems with faster-acting components, such as riboswitches, may improve the overall performance and adaptability.^4^

Dynamic control strategies are thus increasingly recognized as essential in addressing several core challenges in metabolic engineering and synthetic biology. While traditional static designs optimize gene expression or enzyme activity for a single set of conditions, biological systems and their operating environments are inherently dynamic. For example, during large-scale bioproduction, cells are subject to fluctuating nutrient levels, pH, and oxygen availability despite attempts to tightly regulate reactor conditions.^5^ These variations can lead to reduced productivity or pathway imbalances. Dynamic control enables engineered systems to sense environmental or intracellular changes and adjust pathway fluxes accordingly, improving the robustness and yield.^6,7^ In therapeutic contexts, such as engineered probiotics or cell-based therapies, dynamic regulation is even more critical. Here, genetic circuits must respond precisely to disease-relevant signals, control therapeutic output temporally, and, in some cases, incorporate memory features (e.g., hysteresis) to maintain therapeutic effects beyond transient stimuli.^8^ Thus, integrating dynamic control into synthetic pathways expands the design space to address real-world complexity across industrial and clinical applications.^9^

Standardizing biosensor evaluation criteria, particularly concerning dynamic performance, is critical to facilitating their broader application in complex biological systems. Addressing challenges such as noise, response times, and output versatility will unlock unprecedented potential in synthetic sensing technologies, enabling the precise control of cellular behavior for industrial and medical applications.^10^

In this perspective, we highlight the advancements in biosensor development and their integration into metabolic engineering and synthetic biology. By bridging natural sensing mechanisms with cutting-edge technologies, biosensors are poised to become a cornerstone of the bioeconomy, enabling precision and scalability in diverse applications from diagnostics to industrial biomanufacturing.

## Types of Biosensors

To enhance microbial engineering for improved production yields, genetic biosensors that couple metabolite concentrations to measurable or actionable outputs have emerged as indispensable tools across biomanufacturing, diagnostics, and environmental monitoring.^11^ These biosensors generally fall into two categories: protein-based sensors and RNA-based sensors (Table 1). Each category offers unique sensing principles and application strengths.

**Table 1:** Types of Biosensors

category	biosensor type	sensing principle	response characteristics	advantages
protein-based	transcription factors (TFs)	ligand binding induces DNA interaction to regulate gene expression	moderate sensitivity; direct gene regulation	suitable for high throughput screening; broad analyte range
protein-based	two-component systems (TCSs)	sensor kinase autophosphorylates and transfers signal to response regulator	high adaptability; environmental signal detection	modular signaling; applicable in varied environments
protein-based	GPCRs	ligand binding activates intracellular G-proteins and downstream pathways	high sensitivity; complex signal amplification	widely tunable; compatible with eukaryotic systems
protein-based	enzyme-based sensors	substrate-specific catalytic activity generates a measurable output	high specificity; rapid response	expandable via protein engineering
RNA-based	riboswitches	ligand-induced RNA conformational change affects translation	tunable response; reversible	compact; integrates well into metabolic regulation
RNA-based	toehold switches	base-pairing with trigger RNA activates the translation of downstream genes	high specificity; programmable	enables logic-based pathway control; useful in RNA-level diagnostics and production

Among protein-based biosensors, transcription factors (TFs) are a major class that regulate the gene expression by binding DNA in response to specific metabolites.^12^ By linking TF activity to outputs such as fluorescence, these biosensors allow high-throughput screening of strain libraries to identify variants with optimized metabolite production.^12^ TF-based systems are capable of sensing diverse analytes including alcohols, flavonoids, and organic acids^13–15^ (Figure 1a).

**Figure 1:**
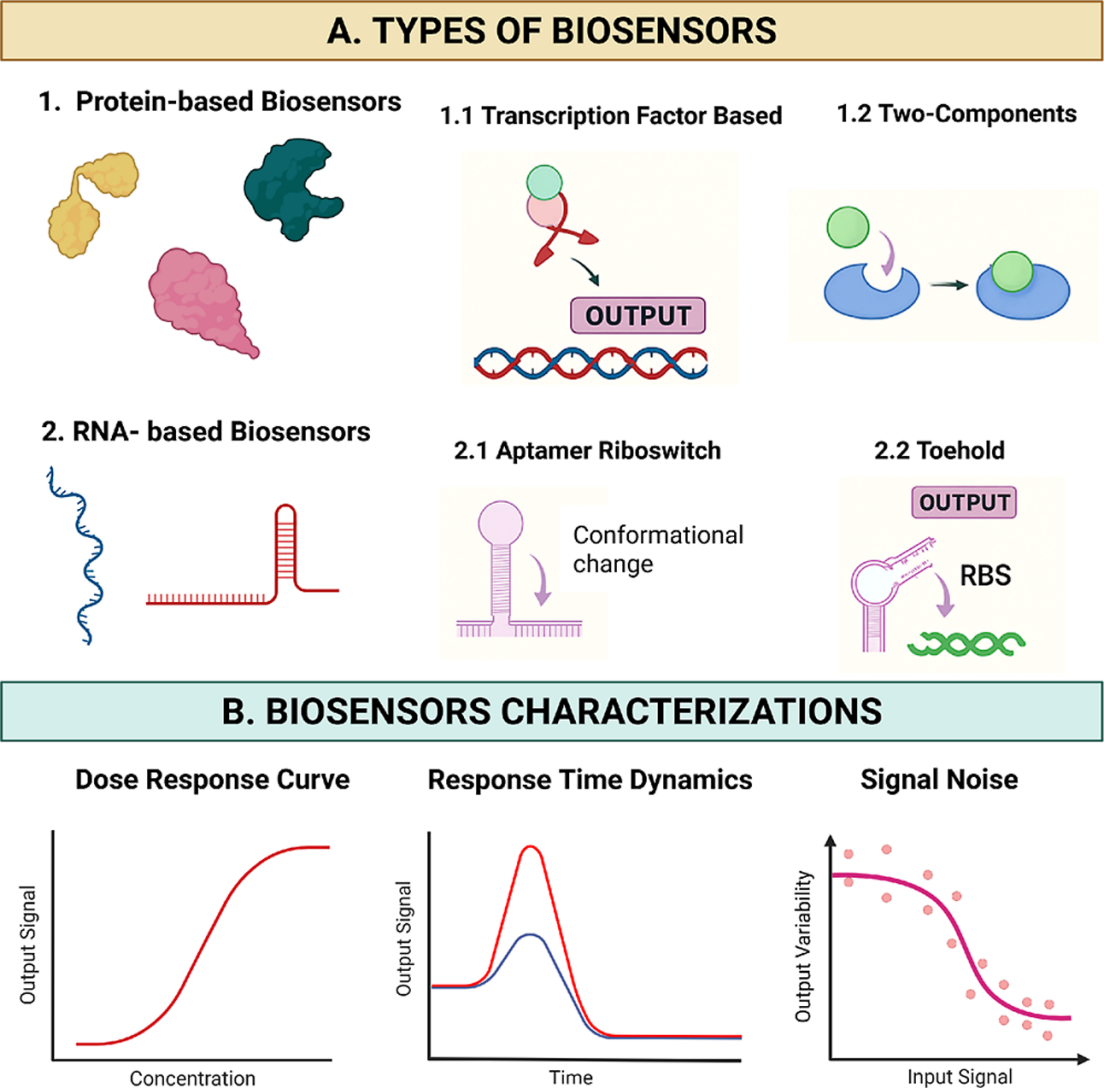
Types and characterizations of biosensors. (A) Biosensors are categorized into protein-based and RNA-based systems. (1.1) Transcription factor-based biosensor, where a small molecule modulates the activity of a transcription factor, leading to gene expression changes that generate an output signal; (1.2) two-component systems utilize signal transduction pathways to detect extracellular or intracellular signals and amplify responses through phosphorylation cascades; (2.1) aptamer riboswitches undergo conformational changes upon ligand binding to control gene expression dynamically; and (2.2) toehold switches are programmable RNA devices that detect complementary nucleic acid sequences and regulate gene expression via ribosome binding site (RBS) accessibility. (B) Key performance parameters for biosensor evaluation include: (i) dose–response curve; (ii) response time dynamics, and (iii) signal noise: represents output variability under a consistent input and affecting biosensor precision and the reliability of high-throughput screening. A biosensor overview with a focus on transcription factor-based biosensors.

Two-component systems (TCSs) and G-protein coupled receptors (GPCRs) extend the functionality of protein-based sensors by enabling cells to detect extracellular or intracellular signals, such as ions, pH, or small molecules, and transduce them through phosphorylation cascades or signal amplification mechanisms.^16,17^ These pathways have been repurposed for real-time environmental and physiological monitoring. In addition, enzyme-based sensors utilize substrate specificity to detect target molecules and can be engineered to expand detection capabilities^18^ (Figure 1a).

On the RNA-based side, riboswitches and toehold switches provide dynamic modular control of gene expression. Riboswitches undergo conformational changes upon ligand binding, enabling the real-time regulation of metabolic fluxes.^19,20^ They are particularly useful for sensing intracellular metabolites, such as nucleotides and amino acids^19,20^ (Figure 1a). Toehold switches, programmable RNA devices that respond to complementary nucleic acid sequences, have been leveraged in microbial engineering to detect intracellular RNA indicators of pathway activity.^19,21^ When integrated into synthetic circuits, toehold switches enable the logic-gated control of metabolic pathways, increasing the production efficiency and enabling precise control over engineered pathways in microbial factories^19,21^ (Figure 1a).

Beyond sensor type and specificity, thorough biosensor characterization is essential to ensure functional reliability and scalability. Critical performance parameters include the dose–response curve, which defines the sensor’s sensitivity and dynamic range by mapping the output signal as a function of analyte concentration. An optimized dose–response curve ensures that the biosensor operates within a useful detection window for the desired metabolite concentrations.^22^ Additionally, response time dynamics, which describe how quickly a biosensor reaches its maximum signal after exposure to the target, are pivotal for applications requiring rapid decision-making or real-time monitoring.^23,24^ Slow or delayed response times may limit the biosensor utility in dynamic environments. Signal noise, another key consideration, reflects the variability of the output signal under constant input conditions.^25,26^ High noise levels can obscure subtle differences in metabolite concentrations, reducing the sensor’s resolution and complicating high-throughput screening workflows (Figure 1b).

Despite the significant progress in repurposing native biosensing systems, their de novo design, fine-tuning of interaction properties, and integration into synthetic circuits remain active areas of research. Current challenges limiting their broader applications include the limited availability of orthogonal or modular sensing elements, which constrains the diversity of detectable compounds, and the technical variability in engineering methods, such as the context-dependent performance and difficulties in tuning sensor sensitivity or dynamic range. Furthermore, biosensors with nonideal dose–response characteristics, sluggish response dynamics, or high signal noise may exacerbate scalability challenges by increasing false positives or masking true high-performing strains during screening.^22–24^ Additionally, the scalability for high-throughput strain engineering remains a bottleneck. This refers to the ability to use biosensors efficiently in workflows involving the screening of large mutant libraries or combinatorial pathway variants—processes that are often constrained by sensor instability, nonlinearity, or cross-reactivity, thereby increasing the resource and time burden in microbial strain development.

### Engineering Biosensors

Even though nature provides numerous examples of biosensors that successfully regulate essential cellular processes, the requirements for biomanufacturing often differ from the natural functionalities of these biosensors. As a result, significant efforts are directed toward enhancing biosensor responsiveness to better align with the needs of metabolic engineering and biomanufacturing. Assessing the behavior of biosensors is often based on the parameters that characterize their response and influence their outcome, depending on their applications.^27^ Typically, biosensor’s response is characterized by its dose–response curve, which can be measured based on the response threshold, dynamic range, and response sensitivity.^27^ Achieving the desired specifications in the biosensor can be made by the selection of the design part, for instance, response sensitivity can be tuned by a plasmid copy number.^28^ However, those parameters are not independent; a trade-off between the dynamic range and biosensor threshold is, for instance, typically found. As biosensors are more often used in regulating the dynamic response, other performance parameters related to dynamic behavior are needed, such as rise-time.^29^ More in general, biosensor behavior can be assessed within the framework of robustness, which is ultimately needed in biomanufacturing applications.^30^

Engineering approaches for tuning the dynamic and operational ranges of the biosensor typically involve exchanging promoters^31^ and ribosome binding site^32^ number and position of the operator region. The chimeric fusion of the DNA and ligand binding domains have also been used to engineer the specificity of the biosensor.^15^ High-throughput techniques like cell sorting, combined with directed evolution strategies, can lead to improved sensitivity and specificity to the engineered biosensor.^33^ Notably, approaches based on the AI-assisted design of transcription factor-based biosensors are increasingly integrated into the engineering pipeline.^34^

Given the modular nature of allosteric transcription factor-based biosensors, their engineering has primarily focused on five key design areas: the DNA-binding domain (DBD), the ligand-binding domain (LBD), the core region of the responsive promoter (transcription factor binding site, TFBS), the operator regions, and the ancillary regulatory components. Rationally tuning any of these areas requires multiple iterations of the design-build-test-learn (DBTL) cycle,^35^ which can be resource-intensive, making it crucial to efficiently navigate the biosensor design space and identify key factors that significantly impact biosensor behavior.^36^ The DBD, which typically binds to the DNA binding site of the responsive promoter via the helix-turn-helix (HTH) DNA binding motif,^37^ interacts with the major groove of DNA through hydrogen bonds and noncovalent interactions. Its position within the transcription factor influences transcriptional regulation mechanisms, with N-terminal DBDs often found in repressors and C-terminal DBDs generally associated with activators.^38^ Biosensors are commonly classified based on DBD conservation using profiles, such as Pfam,^39^ and engineering the DBD allows for transcriptional regulation tuning, as demonstrated by TetR variants with HTH motif mutations.^40^ Similarly, the LBD facilitates allosteric conformational changes upon binding to a small molecule, indirectly altering transcriptional regulation.

Unlike DBDs, LBDs are less conserved, and modifying their specificity typically requires multiple rounds of directed evolution.^41,42^ Engineering the LBD enables ligand specificity tuning, and chimeric transcription factors combining DBD and LBD can be designed for customized regulatory functions.^15,43^ Additionally, modifying the TFBS, including the core promoter and operator regions, as well as engineering regulatory components, such as transcriptional and translational regulation of both the transcription factor and the target genetic circuit, further expands biosensor versatility.^44^ By refining these components, researchers can develop biosensors with optimized sensitivity and specificity for diverse applications in biomanufacturing and metabolic engineering.

### Biosensors High-Throughput Screening

High-throughput screening (HTS) using biosensors has emerged as a cornerstone of synthetic biology, providing a platform for rapid prototyping through single-cell selection and directed evolution.^45^ This approach has enabled the identification and optimization of desirable traits at an unprecedented pace, significantly accelerating advancements in fields, such as metabolic engineering, enzyme discovery, and synthetic circuit design^46,47^ (Figure 2).

**Figure 2:**
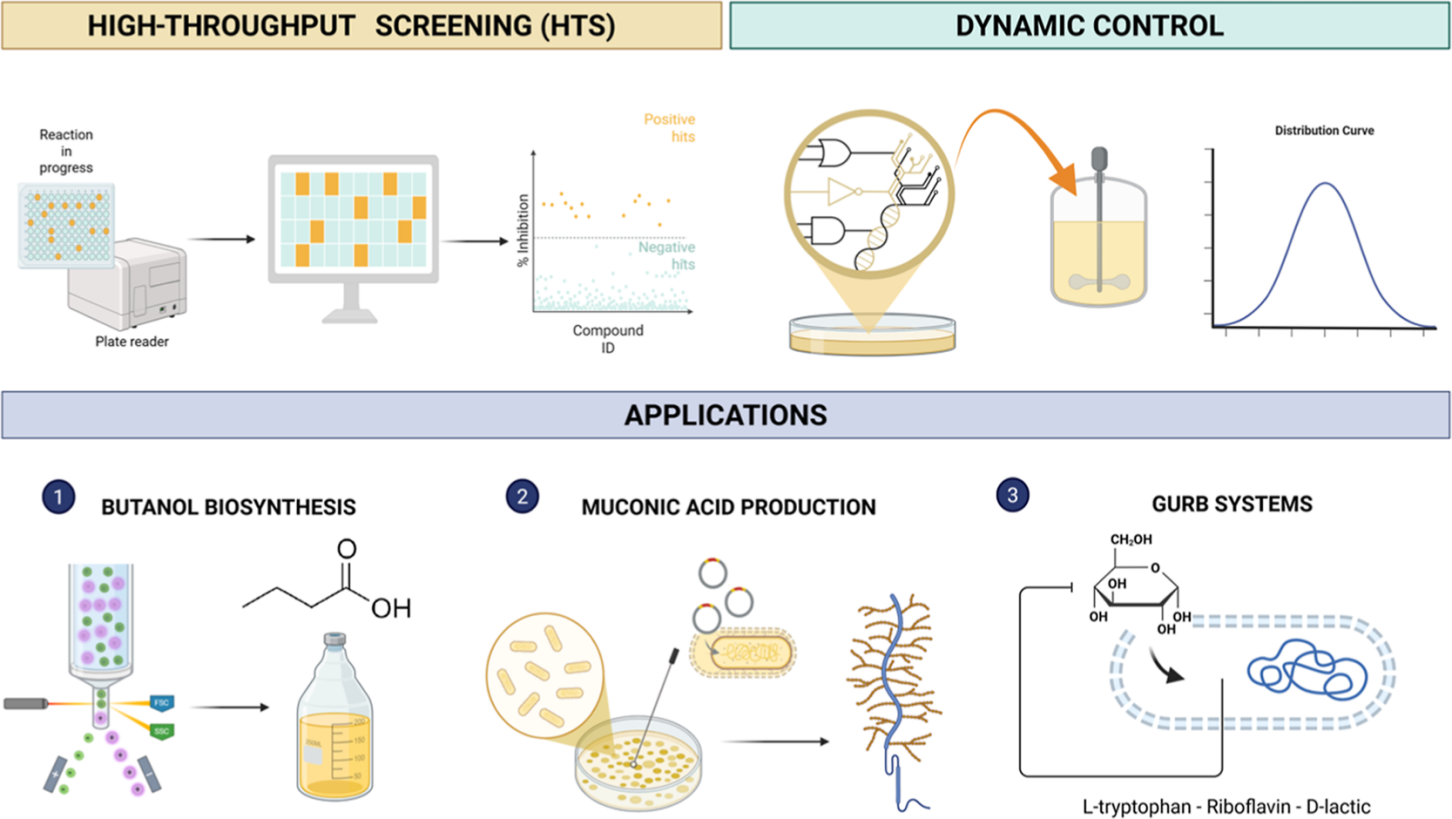
High-Throughput Screening (HTS) and dynamic genetic control in synthetic biology applications. The top left panel illustrates HTS that enables rapid single-cell analysis, selection, and evolution of microbial traits by linking biosensor outputs to measurable readouts (e.g., fluorescence or growth advantage), facilitating the identification of high-performing variants. The top right panel illustrates dynamic control circuits, incorporating sensing, processing, actuation, and feedback, allowing the real-time regulation of the gene expression and metabolic flux to optimize the cellular performance. The bottom panel illustrates some highlighted applications: (1) butanol biosynthesis via FACS and enzyme engineering; (2) muconic acid production through biosensor-linked selection; and (3) glucose uptake rate-based systems that dynamically regulate glucose assimilation to enhance the production of l-tryptophan, riboflavin, and d-lactic acid. Together, these technologies drive the development of sustainable efficient microbial platforms for industrial biotechnology.

Notwithstanding these challenges, HTS has already demonstrated remarkable success stories. For instance, the engineering of microbes for the sustainable production of biofuels and bioplastics has been achieved, along with the discovery of enzymes optimized for industrial applications like bioremediation and pharmaceutical synthesis.^47,48^ Specific HTS-driven breakthroughs include the identification of novel alcohol dehydrogenases for butanol biosynthesis and the evolution of thermostable cellulases for bioethanol production using fluorescence-activated cell sorting (FACS)-based biosensors. Additionally, HTS enabled the selection of strains overproducing muconic acid through genetically encoded sensors linked to antibiotic resistance selection, streamlining the metabolic engineering of shikimate pathways. Genetically encoded biosensors have been transformative tools in these applications, not only enabling the detection of metabolites but also facilitating the screening of large mutant libraries to uncover rare high-performing variants.

However, despite its transformative potential, HTS faces notable limitations. Biosensors often lack specificity, leading to false positives or negatives in complex environments, and their performance can be inconsistent when applied to challenging biological contexts.^46^ These issues highlight the need for more robust and reliable biosensor designs. The field is now progressing toward addressing existing challenges through innovations. These advancements are expected to enhance both the throughput and accuracy of HTS, strengthening its role as a key tool in biotechnology. Recent innovations, such as programmable bifunctional biosensors that integrate real-time monitoring with the dynamic regulation of metabolic pathways, further underscore the transformative potential of HTS.^35,49^

### Dynamic Genetic Control Systems for Adaptive Regulation

Parallel to HTS, dynamic genetic control systems are revolutionizing the regulation and optimization of cellular behavior. These systems allow real-time adjustments to metabolic pathways, enabling cells to adapt to changing environmental conditions or resource availability while maintaining optimal performance.^50^

The principles of dynamic genetic control can be schematized in (1) sensing module: detects intracellular or extracellular signals (e.g., metabolite levels, pH, temperature, light); (2) processing module: interprets the signal using regulatory logic (e.g., synthetic promoters, riboswitches); (3) actuator module: modifies gene expression in response (e.g., increasing enzyme levels, reducing transport proteins); and (4) feedback loop: adjusts signal strength or expression rates based on real-time measurements, ensuring homeostasis. This architecture enables cells to self-regulate biosynthetic pathways, detecting deviations and activating countermeasures. Dynamic regulation has proven to be particularly valuable in improving the yield of complex bioproducts and reducing the metabolic burden on host organisms.

For example, glucose uptake rate biosensors, such as the glucose uptake rate based (GURB) system, have been designed to monitor and dynamically adjust glucose assimilation in real time, addressing critical bottlenecks in metabolic flux optimization. These biosensors have successfully improved the production of bioproducts like l-tryptophan, riboflavin, and d-lactic acid by balancing metabolic fluxes and reducing byproduct accumulation.^51^

However, achieving absolute precision in the genetic control remains a significant challenge due to the inherent complexity of cellular networks and the unpredictable interplay of metabolic and regulatory pathways.^52,53^ Despite these hurdles, researchers continue to push the boundaries of innovation, exploring inducible gene circuits, synthetic feedback loops, and adaptive regulation strategies.^54^

In addition to these advancements, recent studies have explored the integration of electrothermal processes in synthetic biology applications. For instance, the development of electrothermal chlorination techniques has enabled the rapid and selective recovery of critical metals from electronic waste, highlighting the potential of combining electrical and thermal inputs for efficient bioprocessing.^54^ This approach not only enhances the sustainability of biotechnological processes but also opens new avenues for resource recovery and recycling.

Furthermore, the design of biologically sourced depolymerizable polymers with intrinsically weakened carbon–carbon bonds has been achieved, facilitating controlled chemical recycling to monomers.^54^ These polymers, synthesized via simple free-radical polymerization, demonstrate the potential of integrating synthetic biology with materials science to create sustainable and recyclable materials.

As synthetic biology continues to evolve, the integration of novel materials and processes, such as those mentioned above, will play a crucial role in addressing global challenges. The convergence of HTS, dynamic genetic control, and innovative material design holds the promise of revolutionizing industries ranging from healthcare to environmental management, paving the way for a more sustainable and prosperous future.

Looking ahead, the convergence of HTS and dynamic genetic control holds immense promise. By combining the high-throughput capabilities of biosensors with the precision and adaptability of genetic regulation, future innovations could revolutionize synthetic biology. Potential breakthroughs include the creation of ultraefficient microbial cell factories, the rapid design of therapeutic solutions, and the development of more sustainable processes for agriculture and industry.^55^ Together, these tools are poised to address some of the most pressing global challenges, from environmental sustainability to human health, and their ongoing evolution is likely to define the next era of biotechnological innovation.

### Biosensors for Computer-In-The-Loop Advanced Control

The outputs from allosteric transcription factor-based biosensors provide valuable insights into the internal states of the cell, such as the intracellular concentration of key metabolites or physiological states of the cell, such as stress response or nutrient depletion. This time-varying data flow can be leveraged in multiple ways. Within the cell, biosensors can be used to establish feedback loops to activate dynamic regulation circuits. However, possibilities are greatly enlarged when biosensor readouts are extracted for external processing, for instance by expressing a fluorescent, luminescent, or colorimetric reporter, or through electrogenicity.^56^ This enables the implementation of computer-in-the-loop strategies (Figure 3), eventually providing access to a wide array of data-driven feedback algorithms for advanced control and analysis.^57^ Moreover, biosensor data can be combined with other data sources and analytical techniques, for instance, single-cell analysis from flow cytometry.^58^ Once the data has been processed by the computing algorithms, the control signals need to reach the cell by converting them through actuation signals, for instance, optogenetics.^59^ In a similar fashion, biosensor signals can be used for process control during fermentation. For instance, they can be combined with single-cell analysis in microbial production strains to monitor the physiological state of the cells, such as nutritional state or cofactor supply,^60^ and develop strategies for the optimization of the bioprocess.^61^

**Figure 3:**
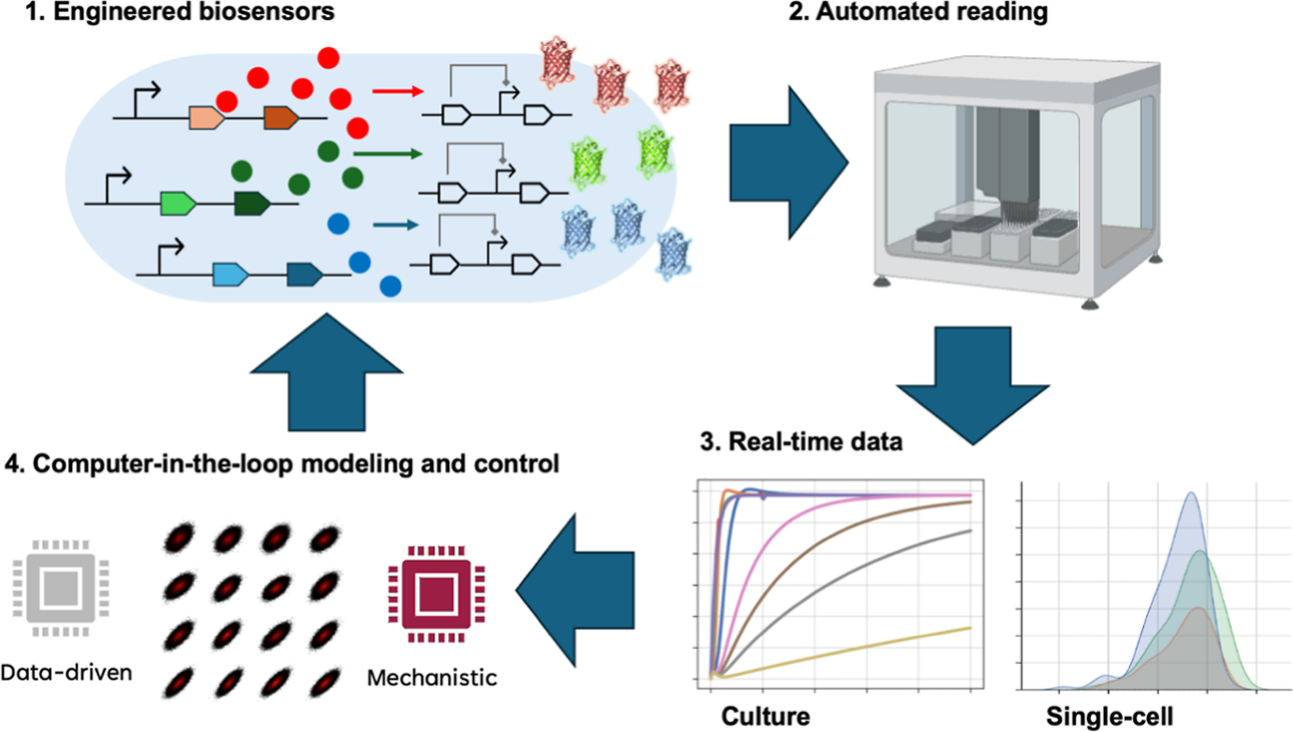
Biosensor integration into computer-in-the-loop strategies. Engineered biosensors reporting at key nodes in biomanufacturing strains (1) generate real-time data that can be acquired in automated reading systems (2). The data can be either measured at culture or single-cell domains, providing in each case readouts representing distinct levels of cellular dynamic states (3). A computer-in-the-loop algorithm implementing advanced data processing (4) can be designed to combine mechanistic prior models with data-driven approaches, such as extended dynamic decomposition and deep learning, to elicit actuating signals for the precise control of the producing pathways.

In computer-in-the-loop biosensing, two broad categories of dynamic control strategies exist: model-based and data-driven approaches. A first class of strategies relies on model-based control, where biosensor readouts are compared against reference values, and an inverse model is used to compute the actuation law.^62^ For instance, model predictive control (MPC), based on predicting the future behavior of the system based on a finite prediction horizon, has been proposed for synthetic biology applications, enabling precise regulation in some cases for protein production applications.^63^ However, model-based approaches face significant challenges in synthetic biology. A key limitation is the need for an accurate model of the underlying system, which is often unavailable due to the complexity of genetic networks and cellular processes. Additionally, unmodeled dynamics—including noise, metabolic burden, and context-dependent effects—can degrade the control performance, limiting the robustness of these strategies.^57^

A second category of strategies leverages data-driven approaches, where the system’s dynamic behavior is inferred directly from biosensor readouts. This can be achieved through adaptive control methods that iteratively estimate an approximate linear model of the system.^64^ More advanced techniques involve machine learning, which can extract patterns from time-series data and adapt control strategies accordingly.^65^ However, data-driven approaches face the underlying challenge of limited biological interpretability. Even though hybrid frameworks based on biology-guided machine learning models have been proposed,^66^ a major issue arises from the coverage of the data. High volumes of experimental data covering a wide array of perturbations are needed by the learning algorithm to perform accurate inference. A particularly promising direction involves nonlinear transformations of biosensor signals, inspired by techniques, such as Extended Dynamic Mode Decomposition (EDMD) and Koopman operator theory.^67^ These methods enable a higher-dimensional representation of system dynamics, facilitating the prediction and control of complex biological networks. Hybrid approaches that integrate mechanistic models with deep learning architectures are gaining traction as well, providing a flexible framework that balances interpretability and predictive power.^68^ Such hybrid representations offer the potential to bridge the gap between the traditional control theory and data-driven insights, paving the way for more robust and scalable synthetic biology applications.

### Challenges and Future Directions

Achieving high performance, robustness, and reproducibility in biosensors applications for smart biomanufacturing will ultimately depend on addressing the implementation challenges that are currently preventing their broader adoption. Among those implementation challenges, cross-talk between biosensors and host metabolism can be mentioned. First, regarding the specificity of the DBDs in the sensor, several strategies are possible to enhance their DNA binding specificity DBD, avoiding cross-talk of the transcription factor with multiple genetic sites.^69,70^ More interestingly, the cross-talk of the LBD of the transcription factor with other effectors or with metabolites analogue to the biosensor target is often found in applications, and several strategies have been proposed to address it. For instance, Marionette is a collection of *Escherichia coli* strains containing 12 biosensors that were selected and optimized to avoid cross-talk.^71^ The collection of biosensors was developed through a dual-selection directed evolution scheme for an improved dynamic range and decreased cross-talk. Implementation challenges for biosensors are also associated with their industrial scalability. Biosensors are often designed under laboratory conditions for the desired dynamic range. However, the responses of different biosensors can have different growth dependences. Modeling such dependencies can provide the ability for predicting biosensor behavior in a broader set of conditions.^72^

In conclusion, we showed here that biosensors are a key component in the current development in fields, such as smart bioprospecting, bioremediation, and biomanufacturing. We might envision some future directions in the field. A higher throughput systems for biosensor development,^73^ combined with machine learning and AI techniques;^34,74^ more powerful computational techniques exploring the biosensor design space;^75,76^ a higher integration of biosensors with robotics platforms;^77^ more accurate calibration of biosensors;^44^ improved pathway dynamic regulation;^78,79^ single-cell biosensor analysis;^80^ as well as an enlarged portfolio of terrestrial, marine, and airborne biosensors, environmentally deployed^81^ and for space applications.
